# Impact of Immunosenescence on Vaccine Immune Responses and Countermeasures

**DOI:** 10.3390/vaccines12111289

**Published:** 2024-11-19

**Authors:** Li Chen, Chengwei Shao, Jingxin Li, Fengcai Zhu

**Affiliations:** 1 School of Public Health, Southeast University, Nanjing 210096, China; 220223621@seu.edu.cn (L.C.); 230239097@seu.edu.cn (C.S.); 2 Jiangsu Provincial Medical Innovation Center, National Health Commission Key Laboratory of Enteric Pathogenic Microbiology, Jiangsu Provincial Center for Disease Control and Prevention, Nanjing 210009, China

**Keywords:** aging, immunosenescence, vaccination, immune response

## Abstract

The biological progression of aging encompasses complex physiological processes. As individuals grow older, their physiological functions gradually decline, including compromised immune responses, leading to immunosenescence. Immunosenescence significantly elevates disease susceptibility and severity in older populations while concurrently compromising vaccine-induced immune responses. This comprehensive review aims to elucidate the implications of immunosenescence for vaccine-induced immunity and facilitate the development of optimized vaccination strategies for geriatric populations, with specific focus on COVID-19, influenza, pneumococcal, herpes zoster, and respiratory syncytial virus (RSV) vaccines. This review further elucidates the relationship between immunosenescence and vaccine-induced immunity. This review presents a systematic evaluation of intervention strategies designed to enhance vaccine responses in older populations, encompassing adjuvant utilization, antigen doses, vaccination frequency modification, inflammatory response modulation, and lifestyle interventions, including physical activity and nutritional modifications. These strategies are explored for their potential to improve current vaccine efficacy and inform the development of next-generation vaccines for geriatric populations.

## 1. Introduction

The global shift toward an aging population has resulted in a marked proliferation of geriatric disorders, establishing aging as a central focus of contemporary research. Aging is associated with physiological and pathological changes, including a functional decline in multiple tissues and organs, leading to immunosenescence [1]. Immunosenescence, defined as the progressive deterioration of immunological competence throughout the aging process [2], is a hallmark of aging characterized by thymic involution, epigenetic alterations, chronic inflammation, oxidative stress, mitochondrial dysfunction, an imbalance in the naïve-to-memory cell ratio, and dysregulated metabolism [3]. These changes result in attenuated natural killer (NK) cell-mediated cytolysis, compromised dendritic cell function, a diminished pool of virgin lymphocytes, and an expanded population of memory and terminally differentiated lymphocytes, which are particularly pronounced in older populations [4].

Epigenetic alterations play a crucial role in immunosenescence. Over time, significant changes in DNA methylation within immune cells reduce the transcription of genes essential for immune function. For instance, Lim et al. [5] demonstrated that DNA methylation changes serve as robust biomarkers of biological age and correlate strongly with declining immune function. Age-dependent changes in histone acetylation and methylation affect chromatin structure and gene expression, thereby influencing immune cell activation and proliferative capacity [1]. Mitochondrial dysfunction is another key factor in immunosenescence. As primary cellular energy generators, mitochondrial impairment disrupts energy metabolism and elevates oxidative stress. Research has shown that mitochondrial DNA mutations and functional deficits are strongly associated with compromised immune cell functionality [6].

Chronic inflammation is a hallmark of immunosenescence, characterized by persistent low-grade inflammation. Current research shows that elevated levels of pro-inflammatory cytokines, specifically IL-6 and TNF-α, observed in geriatric populations contribute to maintaining chronic inflammation, thereby promoting inflammatory cell activation and migration, which leads to compromised tissue and organ function [7]. Notably, decreased IL-6 levels result in reduced acute-phase protein synthesis, limited T-cell proliferation and activation, and impaired B-cell differentiation into plasma cells, thereby diminishing antibody production [8]. Similarly, reduced TNF levels attenuate the initiation of inflammatory responses, decrease immune cell chemotaxis and recruitment, and impair macrophage phagocytic capacity, potentially increasing susceptibility to infections [9]. Additionally, research has shown that age-associated aberrant immune cell activation exacerbates inflammatory responses [10]. Immune cells, particularly macrophages and neutrophils, may remain persistently activated, releasing increased inflammatory mediators and further amplifying inflammation. Immunosenescence induces a range of metabolic and functional abnormalities in neutrophils, including decreased phagocytic capacity, impaired adhesion and chemotaxis, increased apoptosis, and reduced Toll-like receptor function [11]. Research indicates that neutrophil chemotaxis diminishes with age, resulting in reduced directional migration toward stimuli, including vaccine injection sites [12]. Comparative analyses reveal that patients over 60 years old show significantly reduced neutrophil migration accuracy during severe pulmonary infections compared to those under 35 [13]. Senescence-associated alterations in macrophage functionality encompass various domains, including diminished phagocytic capacity and impaired regulation of the transition between M1 (pro-inflammatory) and M2 (anti-inflammatory) activation states [14]. Consequently, age-associated macrophage dysfunction impairs the resolution of inflammation, further compromising the capacity of other immune cells to mount effective responses against infections or generate protective immunity following vaccination.

During immunosenescence, immune cell dysfunction, particularly the impairment of T and B lymphocytes, is a critical determinant. Aging T cells exhibit features such as diminished proliferative capacity, reduced cytotoxicity, and decreased cytokine secretion. Wherry et al. [15] have shown that T cell exhaustion originates primarily from alterations in antigen recognition-induced signal cascades and diminished synthesis of costimulatory molecules. Experimental evidence indicates that this immunological decline is significantly associated with reduced B-cell genesis and substantial impairment of memory B-cell function [16].

Immunosenescence is a complex, multifaceted process driven by diverse molecular mechanisms. Compared with younger individuals, older adults experience progressive declines in organ function and immune competence, increasing their susceptibility to infectious diseases. This increased susceptibility exacerbates pre-existing conditions, often resulting in severe cases and significantly reducing the functional independence of older adults. Therefore, preventing infectious diseases is essential for maintaining geriatric physiological homeostasis and reducing the associated societal and economic burdens.

In the context of SARS-CoV-2 infection, the geriatric population demonstrates markedly elevated susceptibility and case-fatality rates. Individuals aged 60 and above, particularly those with underlying comorbidities, exhibit a heightened propensity for critical SARS-CoV-2 manifestations, with over 80% of virus-associated mortality occurring in those in advanced geriatric older individuals [17]. The COVID-19 vaccination has been shown to significantly mitigate these risks [18]. Moreover, the geriatric population demonstrates elevated susceptibility to various pathogens, including influenza, pneumococcal disease, herpes zoster (HZ), and respiratory syncytial virus (RSV), all of which impose a substantial disease burden on this population. Influenza-associated case fatality rates demonstrate a positive correlation with advancing chronological age. Iuliano et al. reported that annual global influenza-associated respiratory deaths range from 2.9 × 10⁵ to 6.5 × 10⁵, with peak case fatality indices documented among the advanced septuagenarian demographic [19]. In 2022–2023, influenza mortality rates among individuals in the U.S. geriatric population exceeding 65 years demonstrated more than three times higher than those in other age groups, with 26.6 deaths per 100,000 people, accounting for 72% of total influenza-related deaths, demonstrating the heightened vulnerability of this age group to influenza [20]. Although RSV infection mortality rates are lower than those of influenza, it remains a significant health threat for older adults, particularly those over 80, resulting in 60,000 to 160,000 annual hospitalizations within the U.S. geriatric demographic exceeding age 65 [21]. Furthermore, immunosenescence-associated alterations elevate the susceptibility to pneumococcal infection in older adults [22], with pneumococcal disease mortality rates positively correlated with age in adults over 50 [23]. Vaccination is a crucial medical intervention for preventing infections, hospitalizations, and related complications. It effectively reduces mortality, enhances public health outcomes, and is highly cost-effective.

Currently, most countries recommend annual influenza and pneumococcal vaccinations for the geriatric population exceeding age 65. Additionally, this demographic constitutes a key target cohort for SARS-CoV-2 and herpes zoster vaccinations. In 2023, a significant milestone in vaccinology was reached with the approval of the inaugural RSV immunization formulated for the geriatric aged 60 and older, aimed at mitigating RSV-induced lower respiratory tract disease. This analysis investigates how age-related immune system deterioration modulates vaccination-elicited immunological responses to COVID-19, influenza, pneumococcal, herpes zoster, and RSV vaccines in older adults. This review systematically examines how aging affects immune responses to these vaccines, which are widely administered in this population. It also explores strategies and interventions aimed at enhancing immune responses and improving vaccine efficacy in older adults, providing insights for both optimizing responses to existing vaccines and developing new vaccines or vaccination strategies.

## 2. The Impact of Immunosenescence on Vaccine Immune Responses

### 2.1. COVID-19 Vaccines

Current immunoprophylactic agents targeting SARS-CoV-2 comprise inactivated, live attenuated, viral vector, protein subunit, nucleic acid, and virus-like particle (VLP) formulations [24]. The comparison between the vaccines is shown in Table 1. A long-term immunological response assessment of the CoronaVac inactivated vaccine involving 12,396 participants revealed that following vaccination, soluble immune mediators, including IL-6, TNF-α/β, MIP-β, and IFN-γ, showed a negative correlation with increasing age, likely associated with immunosenescence but independent of the heightened chronic inflammatory state commonly observed in older individuals [25]. Older adults achieved significant levels of binding immunoglobulins specific to the wild-type (WT) strain and its emerging mutants of concern (VOCs) later than younger individuals, and the antibody levels remained consistently lower and declined more rapidly. This may be related to the reduced frequency and responsiveness of antigen-inexperienced lymphocytes among older adults [25]. Studies on BBIBP-CorV support these findings, showing that individuals over 60 exhibited lower antibody levels and delayed antibody production kinetics [26]. Despite age-related declines in immune function, the whole virion β-propiolactone inactivated vaccine CoviVac (produced by Chumakov FSC R&D IBP RAS) demonstrated a seroconversion rate exceeding 85% in both the 18–60 age group and those over 60. The humoral immune response in individuals aged over 60 did not show significant attenuation, with comparable seroconversion rates between age groups (z = 5.180, *p* < 0.001) [27], potentially influenced by the relatively small sample size.

Neutralizing antibody titers and T-cell responses to mRNA vaccines also show a significant age-related decline. Research on BNT162b2 found that participants’ age was negatively correlated with both S glycoprotein-specific antibodies and neutralizing antibody responses [28], with neutralizing antibody titers having a lower age-dependent decline across the adult age spectrum [29]. Two weeks following completion of the second dose of the BNT162b2 vaccine, 19% of older participants (median age 76 years) had neutralizing antibody titers near the lower limit of detection, compared to only 1.4% in the younger group (median age 48) [30]. Demaret et al. compared older participants (mean age 86) with younger participants (mean age 44) at the two-month post-completion timepoint of their BNT162b2 vaccination regimen, finding that the neutralizing antibody titers in the older group decreased tenfold, accompanied by a marked reduction in spike-reactive T-cell responses [31]. Scola et al. similarly observed that older adults (mean age 86) had significantly lower neutralizing antibody titers than younger adults at both early-term (day 31) and intermediate-term (day 105) after the second dose of BNT162b2 [32]. Further studies on T-cell responses showed that IFN-γ and IL-2 were negatively covariances relative to age (r = -0.49) [33]. These studies consistently indicate that antibody levels induced by the BNT162b2 vaccine are lower in older adults. The SYS6006 mRNA vaccine supports this finding, with geometric mean titers (GMTs) of wild-type (WT) neutralizing antibodies of 232.1 and 130.6 in the adult 20 µg and 30 µg groups, respectively, while the older adults group had GMTs of 48.7 and 66.7 [34]. Additionally, studies on other mRNA vaccines have indicated that advanced age is associated with lower antibody levels and T cell responses, with the frequencies of CD4+ and CD8+ T cells producing spike-specific IL-2, IFN-γ, and IgG negatively correlated with age, likely due to reduced frequencies of naïve T cells in older adults [35].

A real-world multicenter longitudinal study found that both mRNA vaccines (BNT162b2, mRNA-1273) and vector vaccines (Vaxzevria, Ad26. COV2-S) exhibited lower antibody levels in older adults, with significantly lower anti-trimeric spike IgG levels and neutralizing antibody titers compared to younger adults [29]. Research has shown that although age affects both antibody levels and T-cell-mediated immunity elicited through the Vaxzevria vaccine, booster doses can achieve comparable protective effects across all age groups [36]. Age was identified as a negative factor in immune responses to the adenovirus type 5 (Ad5) vector COVID-19 vaccine, with participants aged 55 and above exhibiting significantly lower antibody responses compared to younger participants, as evidenced by reduced RBD-specific ELISA antibodies (*p* = 0.0018), live virus neutralizing antibodies (*p* < 0.0001), and pseudovirus neutralizing antibodies (*p* = 0.046) [37]. Studies also indicate that age influences the immunological profile following Ad26.COV2.S administration. Older individuals (≥65 years) demonstrate slower initial antibody production compared to younger adults (18–55 years), potentially due to reduced germinal center reactions or impaired innate immune responses. Despite slower immune response kinetics in older individuals, booster immunization after three months achieves antibody titers comparable to those in younger adults, indicating effective immunological memory [38].

Age, along with other factors, can influence the immune response to COVID-19 vaccines. Following the administration of a 2-dose COVID-19 mRNA vaccine (BNT162b2 or mRNA-1273), older adults exhibited significantly reduced antibody binding and neutralizing activity, with poorer durability compared to younger adults at 1, 3, and 6 months post-vaccination. One month after one month of a third dose, older adults achieved comparable levels of antibody binding, ACE2 competition, and live virus neutralization to those of younger adults [39]. Further studies on Omicron breakthrough infections revealed that both older and younger adults exhibited similar levels of WT and Omicron-BA.1 neutralizing antibody magnitude and persistence following mRNA vaccine booster immunization [40]. Interestingly, older adults who had not been previously infected with COVID-19 showed a more rapid decline in BA.1-specific neutralizing activity, with 96% unable to detect BA.1 neutralizing antibodies six months later, compared to 56% of younger individuals. However, both age cohorts exhibited comparable intensities of humoral responses between older and young adults in a mixed immune state [40]. These findings are consistent with other studies on mixed immunity [41].

Overall, the immune response levels in older adults following COVID-19 vaccination are inferior to those in younger individuals. This disparity is primarily attributed to alterations in gene expression patterns in immune cells, reduced diversity and magnitude of the immune receptor repertoire, and impaired antigen-specific clonal expansion capacity following vaccination [42]. Naïve B-cell secretion capacity is markedly age-dependent deterioration [43], and the quantity of circulating naïve B-cells exhibits robust predictive value for post-vaccination antibody levels following COVID-19 immunization [44]. Comparative analysis of CD8+ TCR repertoire dynamics revealed age-associated disparities in SARS-CoV-2 epitope recognition, with elderly cohorts demonstrating diminished clonal diversity and attenuated post-vaccination proliferative responses relative to their younger counterparts [45]. This further elucidates the reasons why age impacts immune responses from the perspective of T-cell responses, although more specific underlying mechanisms still require further investigation.

**Table 1:** Age-Related Immunological Response Characteristics Among Different COVID-19 Vaccine Platforms.

Vaccine Type	Specific Product	Age-Related Immunological Response Characteristics	References
Inactivated Vaccines	CoronaVac	Inflammatory markers (IL-6, TNF-α/β, MIP-β, and IFN-γ) negatively correlate across the aging spectrum. Older individuals show delayed antibody responses, lower antibody titers, and faster decline compared to younger adults.	[25]
	BBIBP-CorV	Lower antibody titers were observed in individuals > 60 years. Advanced age correlates with slower antibody production rates.	[26]
	CoviVac	No significant age-related differences were observed; seroconversion rates > 85% in both 18–60 and >60 age groups.	[27]
mRNA Vaccines	BNT162b2	Negative correlation between age and spike protein-binding antibodies and neutralizing antibodies. Older group (86 years) showed a 10-fold decrease in neutralizing antibody titers. IFN-γ and IL-2 negatively correlated with age (r = −0.49).	[30,31,32,33]
	SYS6006	Significantly lower GMTs in the older group compared to adults: younger group: 232.1 (20 µg), 130.6 (30 µg); older group: 48.7 (20 µg), 66.7 (30 µg).	[34]
Viral Vector Vaccines	Ad5-vectored vaccine	Subjects ≥ 55 years showed significantly lower responses compared to younger subjects: RBD-specific ELISA antibodies (*p* = 0.0018), live virus-neutralizing antibodies (*p* < 0.0001), pseudovirus-neutralizing antibodies (*p* = 0.046).	[37]
	Ad26.COV2.S	Subjects ≥ 65 years demonstrated slower initial antibody production; after a booster dose at 3 months, they achieved comparable levels to younger adults.	[38]

### 2.2. Influenza Vaccines

Annual immunization with seasonal influenza vaccines remains paramount among effective strategies for conferring protection against influenza while attenuating the incidence of moderate to severe illness and hospitalizations and inhibiting viral transmission. The current spectrum of approved influenza vaccines encompasses inactivated, recombinant, and live attenuated vaccines, which incorporate antigens derived from H1N1, H3N2, and two distinct influenza B virus strains. These vaccines predominantly elicit antibody-mediated recognition of envelope-associated glycoprotein determinants, specifically hemagglutinin (HA) and neuraminidase (NA) [46]. However, the substantial antigenic variability exhibited by these surface proteins results in vaccine effectiveness rates consistently remaining below 60% [47]. Vaccine-induced protective immunity against influenza has demonstrated age-stratified efficacy, achieving 70–90% protection in immunocompetent adults while diminishing to 30–50% in elderly populations (≥65 years) [48]. Both inactivated and recombinant influenza vaccines demonstrate limited efficacy in generating robust immune responses among individuals aged 65 and older and immunocompromised populations—demographic groups that paradoxically exhibit heightened susceptibility to seasonal and emerging influenza virus infections [19]. Although live attenuated vaccines demonstrate the capacity to induce strong, long-lasting cell-mediated and humoral immune responses, persistent concerns regarding their biological safety continue to present substantial challenges [49].

Contemporary influenza vaccines predominantly incorporate four distinct viral strains characterized as quadrivalent formulations. Initial investigations examining innate immune responses in monocyte populations following trivalent inactivated influenza vaccine (TIV) in young adults (aged 21–30) and geriatric subjects (≥65 years) revealed that older participants manifested reduced IL-6 and TNF-α expression coupled with elevated IL-10 expression post-vaccination, relative to their younger participants [50]. IL-10 exerts anti-inflammatory effects in monocytes, and its elevation may suppress IL-6 and TNF-α production, contributing to the diminished immune response to influenza vaccination observed in older adults [51]. Compared to TIV, the quadrivalent influenza vaccine (QIV), which includes an additional B strain, offers broader protection. A study conducted in India comparing the immune responses to QIV in age cohort (18–60 years) and elderly population (≥61 years) showed that younger adults had higher seroprotection rates, slightly higher seroconversion rates, and GMTs of hemagglutination inhibition (HI) and neutralizing antibodies after vaccination than older adults [52]. Similarly, a study from China on QIV reported that older adults (≥65 years) had lower GMTs for H1N1, H3N2, B/Victoria, and B/Yamagata strains compared to younger adults (<65 years), although seroconversion rates were similar. Statistical analyses revealed an inverse age-dependent relationship in post-vaccination hemagglutination inhibition antibody levels across multiple strains: H1N1 (*p* < 0.0001), B/Victoria (*p* = 0.0037), and B/Yamagata (*p* < 0.0001) [53]. These findings collectively indicate that younger adults consistently demonstrate an immune response to influenza vaccination compared to their geriatric participants. Increasing the antigen dose is one strategy for enhancing the immunogenicity and effectiveness of influenza vaccines in older populations [54]. Notably, clinical evaluations of high-dose influenza vaccines in individuals exceeding 75 years have H1N1 titers across the age spectrum [55].

Recent years have witnessed substantial advancements in the development of universal nucleic acid influenza vaccines. Recent data from the clinical evaluation of the messenger RNA-based seasonal influenza vaccine candidate (mRNA-1010) demonstrated that mRNA-1010 consistently generated superior functional antibody responses against influenza A strains across all dosage levels and age groups compared to standard-dose QIV (Afluria) while maintaining comparable responses to influenza B strains. The GMTs ratios of anti-HI antibodies across all viral strains among participants spanning age cohorts 18–49, 50–64, and ≥65 years [56]. The observed GMT ratios comparing mRNA-1010 to Afluria demonstrated concordance with findings from clinical trials evaluating enhanced influenza vaccines against standard-dose formulations in geriatric populations [57]. This could be attributed to the inherent immunogenic and adjuvant properties of mRNA, which stimulate innate immunity and promote cytokine production, ultimately augmenting and enhancing both cellular and humoral immune responses [58].

### 2.3. Pneumococcal Vaccines

While severe pneumococcal disease manifests across all age groups, mortality rates associated with pneumococcal infections demonstrate a progressive age-dependent increase in adults exceeding 50 years [23]. Geriatric populations (≥65 years) and individuals with underlying comorbidities or immunocompromised conditions exhibit substantially elevated susceptibility to pneumococcal disease and associated complications [59]. In 2015, the Advisory Committee on Immunization Practices (ACIP) issued guidance recommending dual immunization with both the 23-valent pneumococcal polysaccharide vaccine (PPV23; Pneumovax 23) and the 13-valent pneumococcal conjugate vaccine (PCV13; Prevnar 13) for the geriatric population aged ≥65 years. Studies have shown that PCV13 is more effective than PPV23, likely because polysaccharide vaccines elicit T-cell-independent antibody responses, whereas conjugate vaccines induce T-cell-dependent responses to the serotypes contained in the vaccine [59]. In 2021, the approval of additional conjugate vaccines, PCV15 (Vaxneuvance) and PCV20 (Prevnar 20), aimed to expand serotype coverage and simplify pneumococcal immunization for adults [60]. Additionally, higher-valency conjugate vaccines, such as PCV21 (Merck & Co., Kenilworth, NJ, USA), currently under clinical investigation, offer new avenues for preventing pneumococcal infections in older adults [61].

PPSV23, PCV13, PCV15, and PCV20 vaccines induce strong serological responses in most older adults; however, immune responsiveness gradually declines with age. Both PPSV23 (R = −0.47, *p* = 0.047) and PCV13 (R = −0.7, *p* = 0.0026) showed significant age-related reductions in response, with individuals over 70 demonstrating weaker transcriptional responses compared to those aged 65–70. These transcriptional responses are characterized by the upregulation of genes associated with T-cell activation and differentiation, NK cell signaling, and cytokine activity [62]. After PCV13 vaccination, pneumococcal serotype-specific IgG concentrations in older adults were significantly lower at 28 days post-vaccination, especially for certain serotypes (serotype 1: *p* = 0.008, serotype 4: *p* < 0.0001, serotype 6A: *p* = 0.007, serotype 6B: *p* = 0.0002, serotype 23F: *p* = 0.0001), and the number of responsive serotypes decreased with age [63]. Similar findings were observed in a study involving healthy adults over 50 years, where older participants (65–74 years and ≥75 years) exhibited lower serotype-specific opsonophagocytic activity (OPA) geometric mean titers compared to younger participants (50–64 years) following PCV13 and PCV15 vaccination [64]. PCV20 showed a similar trend, with higher OPA geometric mean titers in younger participants [65]. However, a Phase 1 study of the new PCV21 vaccine reported no significant age-related differences in functional antibody responses, as the serotype-specific OPA geometric mean titers were similar between participants aged 20–64 years and those over 65 [61]. The lack of observed age differences may be due to the small sample size, and further large-scale studies are needed to explore the relationship between age and immune responses to PCV21.

### 2.4. Herpes Zoster Vaccines

The reactivation of latent varicella zoster virus (VZV) manifests as herpes zoster, characterized by painful vesicular skin eruptions and associated with potentially debilitating sequelae. Vaccination remains the most effective preventive intervention against HZ (herpes zoster) and its associated complications. Currently, two distinct vaccine formulations have secured regulatory approval for geriatric immunization: a live-attenuated zoster vaccine (ZVL) developed from concentrated pediatric varicella vaccine and an advanced novel dual-administration recombinant zoster vaccine incorporating AS01B-adjuvanted glycoprotein E antigen (RZV, Shingrix). The comparison between the two vaccines is shown in Table 2.

Studies have shown that the efficacy of the ZVL vaccine decreases with increasing age of the recipients. The protective efficacy of ZVL against herpes zoster is 69.8% in subjects aged 50–60, 65.5% in those over 60 years old, and 55.4% in individuals over 70 [66,67]. In long-term efficacy studies lasting up to eight years, the overall protection declined to between 4.1% [68] and 32.8% [69]. Consistent with its efficacy, the immunogenicity of ZVL is also negatively correlated with age. Six weeks after ZVL vaccination, subjects exhibited a significant increase in VZV-specific cell-mediated immunity (INF-γ and responder cell frequency) and VZV antibodies, with a stronger VZV-specific immune response observed in the 60–69 age group compared to those aged ≥70 [70]. Another study also demonstrated an increase in VZV-specific Th1 and cytotoxic T lymphocyte cell (CTL) responses across all age groups following ZVL vaccination. However, with each decade of age, the immune response significantly decreased, with older adults showing more exhaustion and senescence markers in VZV-specific CTL compared to younger adults [71].

The efficacy and durability of RZV are significantly higher than those of ZVL. RZV provides over 90% protection in preventing both acute zoster episodes and subsequent PHN manifestations throughout the age spectrum, including individuals aged 80 and above [72], with its effectiveness remaining above 83% for up to eight years post-vaccination [73]. Consequently, RZV has been recommended as the sole or preferred zoster vaccine in many countries. Preliminary immunological analyses of RZV demonstrated consistent immunogenicity across age cohorts (50–59, 60–69, and ≥70 years), with T-cell-mediated and humoral responses remaining comparable after the two-dose series [74]. However, subsequent research confirmed that the persistence of vaccine-elicited adaptive immune responses to RZV is influenced by the age at vaccination. Several studies have demonstrated a decline in the proportion of gE-specific antibody and CD4+ T cell responders with increasing age (≥70 years), although these immune responses remained significantly above baseline [75,76,77]. Advanced age negatively impacted both the levels and avidity of gE-specific antibodies two years after RZV vaccination, with individuals aged ≥70 having lower gE-specific levels at 24 and 60 months compared to those aged 50–59 [76]. Longitudinal analysis throughout a three-year post-vaccination surveillance period revealed cell-mediated immune responses in subjects aged ≥70 compared to the 50–69 age cohort, accompanied by a general reduction in CD4+ T-cell frequencies [78]. RZV elicited superior antibody responses and avidity profiles compared to ZVL, demonstrating sustained elevation of gE-specific and anti-gp antibody levels throughout a five-year post-vaccination period. Unlike ZVL, studies of RZV have not observed an age-related impact on the kinetics of gE-specific Th1 responses [75,77].

**Table 2:** Comparative Analysis of Two Herpes Zoster Prophylactic Modalities: Live Attenuated (ZVL) versus Subunit Vaccine (RZV).

Characteristics	ZVL	RZV
Vaccine Efficacy % (95% CI)		
50–59 years	69.8 (54.1–80.6) [67]	96.6 (89.6–99.3) [72]
60–69 years	65.5 (51.5–75.5) [66]	97.4 (90.1–99.7) [72]
≥70 years	55.4 (39.9–66.9) [66]	97.9 (87.9–100.0) [72]
Long-term Efficacy% (95% CI)		
Year 1	68.7 (66.3–70.9) [68] or 67.5 (65.4–69.5) [69]	97.7 (93.1–99.5) [73]
Year 2	47.2 (44.1–50.1) [69]	92.7 (86.2–96.6) [73]
Year 3	39.1 (33.8–43.9) [68]	92.4 (85.0–96.6) [73]
Year 6	32.9 (23.1–41.5) [68]	84.9 (70.4–93.1) [73]
Year 7	16.5 (1.4–29.3) [68]	85.3 (71.3–93.3) [73]
Year 8	4.2 (−24.0 to 25.9) [68] or 31.8 (15.1–45.2) [69]	84.1 (64.4–94.0) [73]
Age Effect on Immune Response	VZV-specific cell-mediated immunity decreases with age, significantly lower in ≥70 years compared to 60–69 years (*p* < 0.001) [70]. Advanced age correlates with lower peak VZV-specific Th1 cells (*p* < 0.0001) [71]. VZV-specific Th1 and cytotoxic T cell (CTL) responses decline with increasing age [71].	Anti-gE antibody and anti-gE CD4+ T-cell positive response rates decrease with age [75,76,77]^.^ Anti-gE levels at 2 and 5 years were lower in subjects ≥ 70 years compared to 50–59 years [76]. Cell-mediated immune response rates and CD4+ T-cell frequencies at months 12, 24, and 36 post-dose 2 were lower in subjects ≥ 70 years compared to 50–69 years [78]. No age effect on gE-specific Th1 response kinetics observed in RZV studies [75,77].

CI = Confidence Interval. The pattern of decline in long-term protection is similar across age groups.

### 2.5. Respiratory Syncytial Virus Vaccines

Respiratory system diseases caused by RSV infection are widely recognized as a significant public health issue in both infants and older adults. In high-income countries, individuals aged 60 years and older face a considerable burden of RSV-related illness, underscoring the need for effective prevention in this population [79]. Currently, two RSVPreF3 OA vaccines (Abrysvo, Pfizer and Arexvy, GSK) have been approved for use in older adults. Recently, a third vaccine, mRNA-1345 (Moderna), received approval in the United States for older use [80]. Abrysvo contains RSVPreF3 from both RSV-A and RSV-B, while Arexvy is a monovalent vaccine containing RSVPreF3 from RSV-A only but includes an adjuvant (AS01E) to enhance immunogenicity.

Currently approved RSV vaccines demonstrate significant protective efficacy in older populations. Abrysvo demonstrates protective efficacy against RSV-induced bronchopulmonary manifestations in elderly populations, showing 66.7% effectiveness (with ≥2 symptoms) and 85.7% effectiveness (with ≥3 symptoms), with comparable efficacy across different age groups (60–69, 70–79, ≥80 years) and high-risk populations [81]. Arexvy, enhanced with the AS01E adjuvant, exhibits exceptional efficacy against RSV-induced lower respiratory tract disease, achieving 82.6% effectiveness in the first RSV season and maintaining 67.2% protective efficacy through the second season [82]. The Arexvy vaccine demonstrates robust protection across both 60–69 and 70–79 age groups (>80%), with notably high vaccine efficacy of 93.8% observed in the 70–79 age group, indicating strong protective effects even in older age cohorts [82]. These data indicate that both vaccines provide effective RSV prevention options for older populations; however, the declining protective efficacy observed with Arexvy over time suggests the need for further research into optimal immunization strategies.

Clinical evaluation of the Abrysvo RSVpreF vaccine demonstrated that geriatric recipients (≥65 years) mounted robust immune responses comparable to younger cohorts. The immunogenicity results from the first human clinical trials of Abrysvo showed a significant increase in neutralizing antibodies targeting both viral subtypes, along with a marked rise in specific IgG titers [83]. Immunological responses were comparable between younger and older participants [84]. Subsequent studies confirmed these findings, showing that individuals aged 75 years and above had immunogenicity results consistent with the overall population [85]. Comparable vaccine-induced immunological outcomes were observed across geriatric cohorts following the administration of the Arexvy RSVPreF3 vaccine. Research demonstrated that GMTs significantly increased one-month post-vaccination in all age groups (60–69 years, 70–79 years, and ≥80 years). While humoral immune responses specific to both viral strains demonstrated slightly weaker in individuals aged ≥70 years, the 95% confidence intervals across groups overlapped, and immune responses converged over time [86]. Comparative analysis revealed elevated GMTs in the 50–59 age cohort relative to subjects aged ≥60 years, although this difference did not achieve statistical significance [87]. Two studies on the Arexvy RSVPreF3 vaccine also did not find age-related differences in cellular immune responses, suggesting similar CD4+ T cell response magnitude and kinetics across all age groups, with no evidence of RSVPreF3-specific CD8+ T cell responses [86,87]. Similarly, immunogenicity results for mRNA-1345 did not indicate an age-related effect on immune responses post-vaccination. One month after vaccination, the geometric mean fold rises (GMFR) in neutralizing antibodies for RSV-A and RSV-B ranged from 12.1 to 16.6 and 8.7 to 12.6, respectively. By six months, the GMFR had decreased but remained at a minimum level of 4.1, with a similar trend observed in PreF-binding antibodies. These findings were comparable to those measured in younger adults [88]. Other vaccines that have not yet received approval, such as MVA-BN-RSV [89], mRNA-1777 (V171) [90], and Ad26.RSV.preF [91], similarly exhibited age-independent profiles in vaccine-induced humoral and cellular immunogenicity. These consistent immune responses across age groups further support the conclusion that age has a limited impact on the immunogenicity of RSV vaccines mRNA-1777 (V171) [90] and Ad26.RSV.preF [91].

### 2.6. Other Vaccines

Immunosenescence is a key factor affecting vaccine efficacy, with advancing age generally characterized by attenuated vaccine-induced immunological responses. This decline manifests in several ways, including delayed antibody production, reduced antibody titers, and impaired T-cell-mediated immune responses. Multiple studies have confirmed that these age-related changes in immune response are evident across various vaccine types.

Regarding viral vaccines, studies of the Japanese encephalitis virus (JEV) vaccine demonstrate significant immunological deficits in older populations compared to younger individuals. Research shows that approximately 50% of individuals over 60 years failed to achieve protective antibody levels, compared to only 15% in younger populations [92]. Further analysis of JEV-specific memory T-cell function on day 35 post-vaccination revealed significantly lower production of key effector cytokines IFN-γ and IL-10 in older groups compared to younger cohorts, while IL-2 responses remained similar between age groups, indicating age-related alterations in memory T-cell vaccine responses. Studies of the hepatitis B vaccine further confirm age-related effects on vaccine immune responses. With advancing age, post-vaccination seroconversion rates progressively decline, accompanied by significantly reduced HBsAb titers [93]. Regarding cellular immunity, older subjects demonstrate markedly reduced T-cell-mediated immune responses, including diminished cell proliferation and cytokine production. Specifically, adolescents show >10% higher proportions of IFN-γ, TNF-α, IL-5, IL-4, and IL-2 secretion compared to older individuals [93]. This attenuated immune response may be attributed to immunosenescence-related factors, including thymic involution, impaired T-cell generation and differentiation, and decreased antibody production capacity [94].

Regarding bacterial vaccines, pertussis vaccine studies demonstrate a clear negative correlation between immune response and age. Comparison of immune responses across different age groups (children 7–10 years, adolescents 11–15 years, young adults 20–34 years, and older 60–70 years) revealed the lowest memory B-cell activation levels in older populations [95]. Additionally, tick-borne encephalitis virus (TBEV) vaccine studies confirmed the negative impact of age on anti-TBEV IgG responses [96]. In conclusion, immunosenescence leads to a comprehensive decline in vaccine immune responses through its effects on multiple immune system components. The compromised vaccine efficacy results from functional deficits in both the innate and adaptive immune systems, warranting further investigation into this complex immunoregulatory network and its relationship with aging.

## 3. Strategies to Enhance Immune Responses in Older Adults

### 3.1. Adjuvants

Immunosenescence increases the susceptibility of older adults to various infections and exacerbates their severity while also significantly reducing vaccine immunogenicity and efficacy. Immunological adjuvants serve as critical modulators to augment vaccine efficacy in elderly individuals. For example, the FDA-approved adjuvants MF59 and AS03 are widely used in influenza vaccines. MF59 and AS03 are both classic oil-in-water emulsion adjuvants. MF59 adjuvant has demonstrated potential in eliciting robust IgG and cell-mediated immune responses [97]. MF59 adjuvant exhibits dual functionality in antigen delivery and immune stimulation, capable of promoting immune cell (including monocytes, macrophages, and dendritic cells) release of multiple chemokines (CCL4, CCL2, CCL5, and CXCL8), enhancing immune cell recruitment and antigen uptake, facilitating antigen presentation, and subsequently triggering adaptive immune responses [98]. MF59 primarily boosts humoral immune responses, significantly increasing anti-HA IgG titers and seroconversion rates in older adults [99,100]. In a randomized trial involving individuals aged 75 and above, those who received an influenza subunit vaccine with MF59 had significantly higher seroprotection and seroconversion rates compared to those who received a non-adjuvanted vaccine [101]. Additionally, AS03, a squalene-based adjuvant system employed in influenza vaccine development, has been shown to produce enhanced serological responses in older adults compared to whole-virus vaccines [102]. AS03 adjuvant exhibits similar immunopotentiating effects to MF59, with its mechanism incorporating immunoenhancing components such as α-tocopherol. α-tocopherol modulates the expression of various immune signaling molecules, including chemokines CCL2, CCL3, CXCL1, and cytokines such as IL-6. These immunomodulatory molecules not only facilitate antigen-presenting cell (APC)-mediated antigen acquisition but also increase local inflammatory cell infiltration and draining lymph node activity, thereby prolonging antibody persistence [103,104]. Furthermore, Advax adjuvant has also demonstrated a positive impact on influenza-specific IgM responses, as well as seropositivity against H3N2 and B strains [105].

In studies of RSV vaccines, the adjuvant AS01B was found to enhance immune responses in older adults through expanded populations of functionally active APCs, thereby improving the efficiency of gE antigen presentation [76,78]. Participants receiving the AS01B-adjuvanted RSV vaccine showed CD4+ T-cell responses that were five times higher than those in the non-adjuvanted group. In contrast, cellular and antibody responses in the non-adjuvanted group decreased significantly with age, further demonstrating the crucial role of adjuvants in overcoming immunosenescence [74]. The development of new adjuvants is also progressing. Ganoderma lucidum spore polysaccharide, used as an adjuvant in influenza vaccines for immunized mice, significantly enhanced antibody levels. Compared to the vaccine-only group, the polysaccharide-adjuvanted group showed a marked increase in hemagglutination inhibition antibody titers within 14 days after the second immunization. This adjuvant also promoted the proliferation of T and B lymphocytes, increased CD4+ and CD25+ T-cell levels, reduced CD8+ T-cell proportions, and enhanced the expression of MHCII, CD86+, and CD80+ molecules on dendritic cells while promoting the secretion of cytokines such as IL-4 and TNF-α [106]. Novel lipid nanoparticle (LNP) adjuvant systems demonstrate superior delivery efficiency and immunogenicity in mRNA vaccines [107]. Novel adjuvant combinations based on Toll-like Receptors (TLR) ligands (such as the synergistic effect of TLR7/8 and TLR4 agonists) demonstrate enhanced immune response effects [108]. Artificial intelligence-based adjuvant screening platforms have accelerated the discovery of novel adjuvants, providing new approaches for developing personalized adjuvant strategies targeted to specific populations [109].

Vaccine adjuvants primarily exert their immunoenhancing effects through the engagement of innate immune pattern recognition receptors (PRRs). This mechanism exhibits some non-specificity, as PRRs typically recognize a broad range of pathogen- and danger-associated molecular signatures (PAMPs and DAMPs) [110]. Although different types of adjuvants may tend to induce specific types of immune responses (for example, aluminum adjuvants bias toward Th2 responses, while nucleic acid adjuvants like CpG oligonucleotides favor Th1 responses), they typically activate multiple immune cell subsets and signaling pathways simultaneously [111]. This broad immune activation may trigger the production of inflammatory mediators (such as IL-1β, TNF-α, and IL-6) and immune cell activation, theoretically increasing the risk of autoimmune responses [112]. Additionally, the type of adjuvant and the duration of inflammation it induces significantly influence T-cell fate decisions and the formation of immunological memory [113]. The development of appropriate adjuvants is crucial for enhancing vaccine immunogenicity. In conclusion, adjuvants can mitigate the negative effects of immunosenescence. Future research should focus on developing novel adjuvants while mitigating adjuvant-associated risks, with the aim of further enhancing vaccine immunogenicity in the older adult population.

### 3.2. Increasing Antigen Dosage

Compared with traditional vaccines, high-dose influenza vaccines significantly increase antibody titers in older recipients, enhancing hemagglutination inhibition and antibody neutralization capacity [114]. Currently, the trivalent influenza vaccine for older adults contains four times the standard dose [115]. Verschoor’s study showed that individuals over 65 who received the high-dose influenza vaccine demonstrated enhanced serological responses against A/H1N1, A/H3N2, and B strains. The high-dose vaccine also induced stronger T follicular helper cell responses and increased plasmablast numbers [54]. Similarly, a double-dose PCV13 pneumococcal vaccine regimen showed superior immunogenicity compared to a single dose in adults over 55 [116]. In the COVID-19 mRNA-1273 vaccine trial, older adults receiving a 100 μg dose exhibited stronger antibody and T-cell responses than those receiving a 25 μg dose [117]. These findings suggest that high-dose vaccination effectively enhances humoral immune responses in older adults, providing a strong basis for improving vaccine efficacy and supporting its application in more vaccination programs.

### 3.3. Repeated Vaccination

In addition to increasing vaccine dosages, annual booster vaccinations are crucial for maintaining humoral immunity in older adults, particularly against influenza and COVID-19 [118]. During the COVID-19 pandemic, booster doses significantly enhanced immune responses. Three weeks after the first dose of the BNT162b2, the proportion of individuals over 80 with sufficient neutralizing antibody titers was lower than that of younger adults; however, after the second dose, neutralizing antibody responses were similar across all age groups [33]. Clinical data revealed enhanced immunogenicity of mRNA booster vaccination against WT and Omicron SARS-CoV-2 in elderly individuals [40], and boosters also extended the duration of the antibody response [28]. Heterologous vaccination strategies have also shown promise in enhancing immunity. For instance, receiving an mRNA vaccine after an inactivated vaccine significantly increased RBD-specific memory B cell and S1-specific T cell responses [119], making this approach an effective way to improve immune responses in older adults. In RSV studies, repeated vaccination also improved immune efficacy. After a third dose of the RSV PreF3 OA vaccine, neutralizing antibody titers and median CD4+ T cell frequencies increased significantly, further enhancing immune responses in older adults [120]. For those who received PPSV23 at least five years prior, a repeat dose increased serotype-specific IgG GMCs and OPA GMTs, continuing protection against pneumococcal disease [121]. Similarly, timely booster doses of the tick-borne encephalitis virus vaccine also offer substantial immunological benefits in enhancing immunity in older adults [96].

### 3.4. Other Approaches

Pre-vaccination anti-inflammatory interventions have demonstrated the potential to enhance vaccine-induced immunity in geriatric populations. Specifically, oral administration of p38 MAPK inhibitors before skin challenge with varicella zoster virus antigens suppresses the production of inflammatory factors, thereby improving immune responses [122]. Additionally, topical application of rapamycin before influenza vaccination can increase antibody titers by approximately 20% and reduce the number of PD-1-expressing CD8+ and CD4+ T cells, thereby enhancing the overall immune response [123].

Exercise and diet also positively influence vaccine immune responses. Research has demonstrated that physical exercise can enhance antibody responses to various types of vaccines, including influenza and COVID-19 vaccines [124]. Regular light to moderate exercise can increase antibody levels following inactivated COVID-19 vaccination in older adults [125]. Antibody titers were higher in individuals aged 65–85 who engaged in moderate exercise before vaccination [126]. Additionally, nutritional interventions are potential strategies for modulating immune responses. Lactobacillus DN-114001 probiotics were found to enhance vaccine-induced immunogenicity in elderly populations, with the seroconversion rate for the A/Michigan/2015 strain demonstrating a threefold elevation in seroconversion rates under probiotic supplementation [127]. Similarly, consuming probiotic K8 enhanced specific IgG and IgA antibody levels against COVID-19 in older adults [128].

In conclusion, strategies such as optimizing adjuvants, increasing doses, and incorporating exercise and dietary interventions effectively address the decline in immune responses due to immunosenescence. The combined use of these approaches could significantly improve vaccine efficacy in older adults, enhancing their protection against infectious diseases.

## 4. Conclusions and Outlook

Immunosenescence encompasses multifaceted biological alterations in innate and adaptive immunity, fundamentally affecting vaccine responsiveness in elderly individuals. Elucidating the molecular mechanisms of age-associated immune decline is paramount for developing targeted immunization approaches and enhancing vaccine efficacy in geriatric medicine. Studies have shown that measures such as optimizing adjuvants and increasing vaccine doses can enhance the efficacy of existing vaccines to some extent, partially overcoming the challenges posed by immunosenescence. Nevertheless, current interventions demonstrate inherent constraints in mitigating age-related immunological decline. Therefore, in future vaccine development, a key challenge will be finding ways to effectively address immunosenescence to ensure that vaccines induce higher immune responses in older adults, thereby improving vaccine efficacy. Solving this issue will not only enhance vaccination outcomes for older adults but also have a profound positive impact on public health.

## References

[1] López-Otín C., Blasco M.A., Partridge L., Serrano M., Kroemer G. (2023). Hallmarks of Aging: An Expanding Universe. Cell.

[2] Weiskopf D., Weinberger B., Grubeck-Loebenstein B. (2009). The Aging of the Immune System. Transpl. Int..

[3] Liu Z., Liang Q., Ren Y., Guo C., Ge X., Wang L., Cheng Q., Luo P., Zhang Y., Han X. (2023). Immunosenescence: Molecular Mechanisms and Diseases. Signal Transduct. Target. Ther..

[4] Grubeck-Loebenstein B., Della Bella S., Iorio A.M., Michel J.-P., Pawelec G., Solana R. (2009). Immunosenescence and Vaccine Failure in the Elderly. Aging Clin. Exp. Res..

[5] Lim U., Song M.-A. (2018). DNA Methylation as a Biomarker of Aging in Epidemiologic Studies. Methods Mol. Biol..

[6] Bondy S.C. (2003). Mitochondrial Dysfunction as the Major Basis of Brain Aging. Biomolecules.

[7] Ferrucci L., Fabbri E. (2018). Inflammageing: Chronic Inflammation in Ageing, Cardiovascular Disease, and Frailty. Nat. Rev. Cardiol..

[8] Tanaka T., Narazaki M., Kishimoto T. (2014). IL-6 in Inflammation, Immunity, and Disease. Cold Spring Harb. Perspect. Biol..

[9] Kalliolias G.D., Ivashkiv L.B. (2016). TNF Biology, Pathogenic Mechanisms and Emerging Therapeutic Strategies. Nat. Rev. Rheumatol..

[10] Youm Y.-H., Grant R.W., McCabe L.R., Albarado D.C., Nguyen K.Y., Ravussin A., Pistell P., Newman S., Carter R., Laque A. (2013). Canonical Nlrp3 Inflammasome Links Systemic Low-Grade Inflammation to Functional Decline in Aging. Cell Metab..

[11] Li X., Li C., Zhang W., Wang Y., Qian P., Huang H. (2023). Inflammation and Aging: Signaling Pathways and Intervention Therapies. Signal Transduct. Target. Ther..

[12] Simmons S.R., Tchalla E.Y.I., Bhalla M., Bou Ghanem E.N. (2022). The Age-Driven Decline in Neutrophil Function Contributes to the Reduced Efficacy of the Pneumococcal Conjugate Vaccine in Old Hosts. Front. Cell. Infect. Microbiol..

[13] Sapey E., Patel J.M., Greenwood H.L., Walton G.M., Hazeldine J., Sadhra C., Parekh D., Dancer R.C.A., Nightingale P., Lord J.M. (2017). Pulmonary Infections in the Elderly Lead to Impaired Neutrophil Targeting, Which Is Improved by Simvastatin. Am. J. Respir. Crit. Care Med..

[14] Shaw A.C., Goldstein D.R., Montgomery R.R. (2013). Age-Dependent Dysregulation of Innate Immunity. Nat. Rev. Immunol..

[15] Wherry E.J., Ha S.-J., Kaech S.M., Haining W.N., Sarkar S., Kalia V., Subramaniam S., Blattman J.N., Barber D.L., Ahmed R. (2007). Molecular Signature of CD8+ T Cell Exhaustion during Chronic Viral Infection. Immunity.

[16] Gibson K.L., Wu Y., Barnett Y., Duggan O., Vaughan R., Kondeatis E., Nilsson B., Wikby A., Kipling D., Dunn-Walters D.K. (2009). B-cell Diversity Decreases in Old Age and Is Correlated with Poor Health Status. Aging Cell.

[17] Lithander F.E., Neumann S., Tenison E., Lloyd K., Welsh T.J., Rodrigues J.C.L., Higgins J.P.T., Scourfield L., Christensen H., Haunton V.J. (2020). COVID-19 in Older People: A Rapid Clinical Review. Age Ageing.

[18] Haas E.J., Angulo F.J., McLaughlin J.M., Anis E., Singer S.R., Khan F., Brooks N., Smaja M., Mircus G., Pan K. (2021). Impact and Effectiveness of mRNA BNT162b2 Vaccine against SARS-CoV-2 Infections and COVID-19 Cases, Hospitalisations, and Deaths Following a Nationwide Vaccination Campaign in Israel: An Observational Study Using National Surveillance Data. Lancet.

[19] Iuliano A.D., Roguski K.M., Chang H.H., Muscatello D.J., Palekar R., Tempia S., Cohen C., Gran J.M., Schanzer D., Cowling B.J. (2018). Estimates of Global Seasonal Influenza-Associated Respiratory Mortality: A Modelling Study. Lancet.

[20] CDC Preliminary Estimated Flu Disease Burden 2022–2023 Flu Season. https://www.cdc.gov/flu-burden/php/data-vis/2022-2023.html.

[21] Hamid S., Winn A., Parikh R., Jones J.M., McMorrow M., Prill M.M., Silk B.J., Scobie H.M., Hall A.J. (2007). Seasonality of Respiratory Syncytial Virus—United States, 2017–2023. MMWR Morb. Mortal. Wkly. Rep..

[22] Elias C., Nunes M.C., Saadatian-Elahi M. (2003). Epidemiology of Community-Acquired Pneumonia Caused by Streptococcus Pneumoniae in Older Adults: A Narrative Review. Curr. Opin. Infect. Dis..

[23] Kristensen M., van Lier A., Eilers R., McDonald S.A., Opstelten W., van der Maas N., van der Hoek W., Kretzschmar M.E., Nielen M.M., de Melker H.E. (2016). Burden of Four Vaccine Preventable Diseases in Older Adults. Vaccine.

[24] Li M., Wang H., Tian L., Pang Z., Yang Q., Huang T., Fan J., Song L., Tong Y., Fan H. (2022). COVID-19 Vaccine Development: Milestones, Lessons and Prospects. Signal Transduct. Target. Ther..

[25] Costa P.R., Correia C.A., Marmorato M.P., Dias J.Z.d.C., Thomazella M.V., Cabral da Silva A., de Oliveira A.C.S., Gusmão A.F., Ferrari L., Freitas A.C. (2023). Humoral and Cellular Immune Responses to CoronaVac up to One Year after Vaccination. Front. Immunol..

[26] Xia S., Zhang Y., Wang Y., Wang H., Yang Y., Gao G.F., Tan W., Wu G., Xu M., Lou Z. (2021). Safety and Immunogenicity of an Inactivated SARS-CoV-2 Vaccine, BBIBP-CorV: A Randomised, Double-Blind, Placebo-Controlled, Phase 1/2 Trial. Lancet Infect. Dis..

[27] Gordeychuk I.V., Kozlovskaya L.I., Siniugina A.A., Yagovkina N.V., Kuzubov V.I., Zakharov K.A., Volok V.P., Dodina M.S., Gmyl L.V., Korotina N.A. (2023). Safety and Immunogenicity of Inactivated Whole Virion COVID-19 Vaccine CoviVac in Clinical Trials in 18–60 and 60+ Age Cohorts. Viruses.

[28] Holtkamp C., Schöler L., Anastasiou O.E., Brune B., Fessmann K., Elsner C., Möhlendick B., Čiučiulkaitė I., Dudda M., Trilling M. (2023). Antibody Responses Elicited by mRNA Vaccination in Firefighters Persist Six Months and Correlate Inversely with Age and Directly with BMI. Heliyon.

[29] Fedele G., Trentini F., Schiavoni I., Abrignani S., Antonelli G., Baldo V., Baldovin T., Bandera A., Bonura F., Clerici P. (2022). Evaluation of Humoral and Cellular Response to Four Vaccines against COVID-19 in Different Age Groups: A Longitudinal Study. Front. Immunol..

[30] Canaday D.H., Carias L., Oyebanji O.A., Keresztesy D., Wilk D., Payne M., Aung H., St. Denis K., Lam E.C., Rowley C.F. (2021). Reduced BNT162b2 Messenger RNA Vaccine Response in Severe Acute Respiratory Syndrome Coronavirus 2 (SARS-CoV-2)–Naive Nursing Home Residents. Clin. Infect. Dis..

[31] Demaret J., Corroyer-Simovic B., Alidjinou E.K., Goffard A., Trauet J., Miczek S., Vuotto F., Dendooven A., Huvent-Grelle D., Podvin J. (2021). Impaired Functional T-Cell Response to SARS-CoV-2 After Two Doses of BNT162b2 mRNA Vaccine in Older People. Front. Immunol..

[32] Scola L., Ferraro D., Sanfilippo G.L., De Grazia S., Lio D., Giammanco G.M. (2023). Age and Cytokine Gene Variants Modulate the Immunogenicity and Protective Effect of SARS-CoV-2 mRNA-Based Vaccination. Vaccines.

[33] Collier D.A., Ferreira I.A.T.M., Kotagiri P., Datir R.P., Lim E.Y., Touizer E., Meng B., Abdullahi A., Baker S., Dougan G. (2021). Age-Related Immune Response Heterogeneity to SARS-CoV-2 Vaccine BNT162b2. Nature.

[34] Chen G.-L., Qiu Y.-Z., Wu K.-Q., Wu Y., Wang Y.-H., Zou Y.-Y., Peng C.-G., Zhao J., Su C., Ma J.-H. (2023). Safety and Immunogenicity of Primary Vaccination with a SARS-CoV-2 mRNA Vaccine (SYS6006) in Chinese Participants Aged 18 Years or More: Two Randomized, Observer-Blinded, Placebo-Controlled and Dose-Escalation Phase 1 Clinical Trials. Hum. Vaccines Immunother..

[35] Dudley H.M., O’Mara M., Auma A., Gong J., Ross Y., Gurevich N., Carbone S., Reihs A., Nguyen Y., McComsey G.A. (2023). Rheumatoid Arthritis and Older Age Are Associated with Lower Humoral and Cellular Immune Response to Primary Series COVID-19 mRNA Vaccine. Vaccine.

[36] Swanson P.A., Padilla M., Hoyland W., McGlinchey K., Fields P.A., Bibi S., Faust S.N., McDermott A.B., Lambe T., Pollard A.J. (2021). AZD1222/ChAdOx1 nCoV-19 Vaccination Induces a Polyfunctional Spike Protein–Specific T _H_ 1 Response with a Diverse TCR Repertoire. Sci. Transl. Med..

[37] Zhu F.-C., Guan X.-H., Li Y.-H., Huang J.-Y., Jiang T., Hou L.-H., Li J.-X., Yang B.-F., Wang L., Wang W.-J. (2020). Immunogenicity and Safety of a Recombinant Adenovirus Type-5-Vectored COVID-19 Vaccine in Healthy Adults Aged 18 Years or Older: A Randomised, Double-Blind, Placebo-Controlled, Phase 2 Trial. Lancet.

[38] Le Gars M., Hendriks J., Sadoff J., Ryser M., Struyf F., Douoguih M., Schuitemaker H. (2022). Immunogenicity and Efficacy of Ad26.COV2.S: An Adenoviral Vector–Based COVID-19 Vaccine. Immunol. Rev..

[39] Mwimanzi F., Lapointe H.R., Cheung P.K., Sang Y., Yaseen F., Umviligihozo G., Kalikawe R., Datwani S., Omondi F.H., Burns L. (2022). Older Adults Mount Less Durable Humoral Responses to Two Doses of COVID-19 mRNA Vaccine but Strong Initial Responses to a Third Dose. J. Infect. Dis..

[40] Mwimanzi F., Lapointe H.R., Cheung P.K., Sang Y., Yaseen F., Kalikawe R., Datwani S., Burns L., Young L., Leung V. (2023). Impact of Age and Severe Acute Respiratory Syndrome Coronavirus 2 Breakthrough Infection on Humoral Immune Responses After Three Doses of Coronavirus Disease 2019 mRNA Vaccine. Open Forum Infect. Dis..

[41] Datwani S., Kalikawe R., Mwimanzi F., Speckmaier S., Liang R., Sang Y., Waterworth R., Yaseen F., Lapointe H., Barad E. (2023). Dynamics of T-Cell Responses Following COVID-19 mRNA Vaccination and Breakthrough Infection in Older Adults. Pathog. Immun..

[42] Crooke S.N., Ovsyannikova I.G., Poland G.A., Kennedy R.B. (2019). Immunosenescence and Human Vaccine Immune Responses. Immun. Ageing.

[43] Frasca D., Blomberg B.B. (2020). Aging Induces B Cell Defects and Decreased Antibody Responses to Influenza Infection and Vaccination. Immun. Ageing A.

[44] Schulz E., Hodl I., Forstner P., Hatzl S., Sareban N., Moritz M., Fessler J., Dreo B., Uhl B., Url C. (2021). CD19+IgD+CD27- Naïve B Cells as Predictors of Humoral Response to COVID 19 mRNA Vaccination in Immunocompromised Patients. Front. Immunol..

[45] Xiao C., Ren Z., Zhang B., Mao L., Zhu G., Gao L., Su J., Ye J., Long Z., Zhu Y. (2012). Insufficient Epitope-Specific T Cell Clones Are Responsible for Impaired Cellular Immunity to Inactivated SARS-CoV-2 Vaccine in Older Adults. Nat. Aging.

[46] Krammer F. (2019). The Human Antibody Response to Influenza A Virus Infection and Vaccination. Nat. Rev. Immunol..

[47] Harrington W.N., Kackos C.M., Webby R.J. (2021). The Evolution and Future of Influenza Pandemic Preparedness. Exp. Mol. Med..

[48] Osterholm M.T., Kelley N.S., Sommer A., Belongia E.A. (2012). Efficacy and Effectiveness of Influenza Vaccines: A Systematic Review and Meta-Analysis. Lancet Infect. Dis..

[49] Zhou B., Meliopoulos V.A., Wang W., Lin X., Stucker K.M., Halpin R.A., Stockwell T.B., Schultz-Cherry S., Wentworth D.E. (2016). Reversion of Cold-Adapted Live Attenuated Influenza Vaccine into a Pathogenic Virus. J. Virol..

[50] Mohanty S., Joshi S.R., Ueda I., Wilson J., Blevins T.P., Siconolfi B., Meng H., Devine L., Raddassi K., Tsang S. (2015). Prolonged Proinflammatory Cytokine Production in Monocytes Modulated by Interleukin 10 After Influenza Vaccination in Older Adults. J. Infect. Dis..

[51] Sabat R., Grütz G., Warszawska K., Kirsch S., Witte E., Wolk K., Geginat J. (2010). Biology of Interleukin-10. Cytokine Growth Factor Rev..

[52] Basu I., Agarwal M., Shah V., Shukla V., Naik S., Supe P.D., Srivastava M.K., Giriraja K.V., Pinjar P., Mishra P.K. (2022). Immunogenicity and Safety of Two Quadrivalent Influenza Vaccines in Healthy Adult and Elderly Participants in India—A Phase III, Active-Controlled, Randomized Clinical Study. Hum. Vaccines Immunother..

[53] Xiao T., Wei M., Guo X., Zhang Y., Wang Z., Xia X., Qi X., Wang L., Li X., Leng S.X. (2023). Immunogenicity and Safety of Quadrivalent Influenza Vaccine among Young and Older Adults in Tianjin, China: Implication of Immunosenescence as a Risk Factor. Immun. Ageing.

[54] Verschoor C.P., Belsky D.W., Andrew M.K., Haynes L., Loeb M., Pawelec G., McElhaney J.E., Kuchel G.A. (2022). Advanced Biological Age Is Associated with Improved Antibody Responses in Older High-Dose Influenza Vaccine Recipients over Four Consecutive Seasons. Immun. Ageing.

[55] Shapiro J.R., Li H., Morgan R., Chen Y., Kuo H., Ning X., Shea P., Wu C., Merport K., Saldanha R. (2021). Sex-Specific Effects of Aging on Humoral Immune Responses to Repeated Influenza Vaccination in Older Adults. Npj Vaccines.

[56] Lee I.T., Nachbagauer R., Ensz D., Schwartz H., Carmona L., Schaefers K., Avanesov A., Stadlbauer D., Henry C., Chen R. (2020). Safety and Immunogenicity of a Phase 1/2 Randomized Clinical Trial of a Quadrivalent, mRNA-Based Seasonal Influenza Vaccine (mRNA-1010) in Healthy Adults: Interim Analysis. Nat. Commun..

[57] Cowling B.J., Perera R.A.P.M., Valkenburg S.A., Leung N.H.L., Iuliano A.D., Tam Y.H., Wong J.H.F., Fang V.J., Li A.P.Y., So H.C. (2020). Comparative Immunogenicity of Several Enhanced Influenza Vaccine Options for Older Adults: A Randomized, Controlled Trial. Clin. Infect. Dis..

[58] Qin F., Xia F., Chen H., Cui B., Feng Y., Zhang P., Chen J., Luo M. (2021). A Guide to Nucleic Acid Vaccines in the Prevention and Treatment of Infectious Diseases and Cancers: From Basic Principles to Current Applications. Front. Cell Dev. Biol..

[59] Paradiso P.R. (2012). Pneumococcal Conjugate Vaccine for Adults: A New Paradigm. Clin. Infect. Dis..

[60] Shah A.A. (2022). Simplifying Pneumococcal Immunizations for Adults. Am. Fam. Physician.

[61] Haranaka M., Yono M., Kishino H., Igarashi R., Oshima N., Sawata M., Platt H.L. (2023). Safety, Tolerability, and Immunogenicity of a 21-Valent Pneumococcal Conjugate Vaccine, V116, in Japanese Healthy Adults: A Phase I Study. Hum. Vaccines Immunother..

[62] Ravichandran S., Erra-Diaz F., Karakaslar O.E., Marches R., Kenyon-Pesce L., Rossi R., Chaussabel D., Nehar-Belaid D., LaFon D.C., Pascual V. (2024). Distinct Baseline Immune Characteristics Associated with Responses to Conjugated and Unconjugated Pneumococcal Polysaccharide Vaccines in Older Adults. Nat. Immunol..

[63] van der Heiden M., Shetty S., Bijvank E., Beckers L., Cevirgel A., van Sleen Y., Tcherniaeva I., Ollinger T., Burny W., van Binnendijk R.S. (2024). Multiple Vaccine Comparison in the Same Adults Reveals Vaccine-Specific and Age-Related Humoral Response Patterns: An Open Phase IV Trial. Nat. Commun..

[64] Platt H.L., Cardona J.F., Haranaka M., Schwartz H.I., Narejos Perez S., Dowell A., Chang C.-J., Dagan R., Tamms G.M., Sterling T. (2022). A Phase 3 Trial of Safety, Tolerability, and Immunogenicity of V114, 15-Valent Pneumococcal Conjugate Vaccine, Compared with 13-Valent Pneumococcal Conjugate Vaccine in Adults 50 Years of Age and Older (PNEU-AGE). Vaccine.

[65] Essink B., Sabharwal C., Cannon K., Frenck R., Lal H., Xu X., Sundaraiyer V., Peng Y., Moyer L., Pride M.W. (2022). Pivotal Phase 3 Randomized Clinical Trial of the Safety, Tolerability, and Immunogenicity of 20-Valent Pneumococcal Conjugate Vaccine in Adults Aged ≥18 Years. Clin. Infect. Dis..

[66] Oxman M.N., Levin M.J., Johnson G.R., Schmader K.E., Straus S.E., Gelb L.D., Arbeit R.D., Simberkoff M.S., Gershon A.A., Davis L.E. (2005). A Vaccine to Prevent Herpes Zoster and Postherpetic Neuralgia in Older Adults. N. Engl. J. Med..

[67] Schmader K.E., Levin M.J., Gnann J.W., McNeil S.A., Vesikari T., Betts R.F., Keay S., Stek J.E., Bundick N.D., Su S.-C. (2012). Efficacy, Safety, and Tolerability of Herpes Zoster Vaccine in Persons Aged 50-59 Years. Clin. Infect. Dis..

[68] Tseng H.F., Harpaz R., Luo Y., Hales C.M., Sy L.S., Tartof S.Y., Bialek S., Hechter R.C., Jacobsen S.J. (2016). Declining Effectiveness of Herpes Zoster Vaccine in Adults Aged ≥60 Years. J. Infect. Dis..

[69] Baxter R., Bartlett J., Fireman B., Marks M., Hansen J., Lewis E., Aukes L., Chen Y., Klein N.P., Saddier P. (2018). Long-Term Effectiveness of the Live Zoster Vaccine in Preventing Shingles: A Cohort Study. Am. J. Epidemiol..

[70] Levin M.J., Oxman M.N., Zhang J.H., Johnson G.R., Stanley H., Hayward A.R., Caulfield M.J., Irwin M.R., Smith J.G., Clair J. (2008). Varicella-Zoster Virus–Specific Immune Responses in Elderly Recipients of a Herpes Zoster Vaccine. J. Infect. Dis..

[71] Weinberg A., Pang L., Johnson M.J., Caldas Y., Cho A., Tovar-Salazar A., Canniff J., Schmader K.E., Popmihajlov Z., Levin M.J. (2019). The Effect of Age on the Immunogenicity of the Live Attenuated Zoster Vaccine Is Predicted by Baseline Regulatory T Cells and Varicella-Zoster Virus-Specific T Cell Immunity. J. Virol..

[72] Lal H., Cunningham A.L., Godeaux O., Chlibek R., Diez-Domingo J., Hwang S.-J., Levin M.J., McElhaney J.E., Poder A., Puig-Barberà J. (2015). Efficacy of an Adjuvanted Herpes Zoster Subunit Vaccine in Older Adults. N. Engl. J. Med..

[73] Boutry C., Hastie A., Diez-Domingo J., Tinoco J.C., Yu C.-J., Andrews C., Beytout J., Caso C., Cheng H.-S., Cheong H.J. (2022). The Adjuvanted Recombinant Zoster Vaccine Confers Long-Term Protection Against Herpes Zoster: Interim Results of an Extension Study of the Pivotal Phase 3 Clinical Trials ZOE-50 and ZOE-70. Clin. Infect. Dis..

[74] Chlibek R., Bayas J.M., Collins H., de la Pinta M.L.R., Ledent E., Mols J.F., Heineman T.C. (2013). Safety and Immunogenicity of an AS01-Adjuvanted Varicella-Zoster Virus Subunit Candidate Vaccine Against Herpes Zoster in Adults ≥50 Years of Age. J. Infect. Dis..

[75] Johnson M.J., Liu C., Ghosh D., Lang N., Levin M.J., Weinberg A. (2022). Cell-Mediated Immune Responses After Administration of the Live or the Recombinant Zoster Vaccine: 5-Year Persistence. J. Infect. Dis..

[76] Weinberg A., Scott Schmid D., Leung J., Johnson M.J., Miao C., Levin M.J. (2023). Predictors of 5-Year Persistence of Antibody Responses to Zoster Vaccines. J. Infect. Dis..

[77] Schwarz T.F., Volpe S., Catteau G., Chlibek R., David M.P., Richardus J.H., Lal H., Oostvogels L., Pauksens K., Ravault S. (2018). Persistence of Immune Response to an Adjuvanted Varicella-Zoster Virus Subunit Vaccine for up to Year Nine in Older Adults. Hum. Vaccines Immunother..

[78] Cunningham A.L., Heineman T.C., Lal H., Godeaux O., Chlibek R., Hwang S.-J., McElhaney J.E., Vesikari T., Andrews C., Choi W.S. (2018). Immune Responses to a Recombinant Glycoprotein E Herpes Zoster Vaccine in Adults Aged 50 Years or Older. J. Infect. Dis..

[79] Savic M., Penders Y., Shi T., Branche A., Pirçon J. (2023). Respiratory Syncytial Virus Disease Burden in Adults Aged 60 Years and Older in High-income Countries: A Systematic Literature Review and Meta-analysis. Influenza Other Respir. Viruses.

[80] Mullard A. (2024). FDA Approves mRNA-Based RSV Vaccine. Nat. Rev. Drug Discov..

[81] Walsh E.E., Pérez Marc G., Zareba A.M., Falsey A.R., Jiang Q., Patton M., Polack F.P., Llapur C., Doreski P.A., Ilangovan K. (2023). Efficacy and Safety of a Bivalent RSV Prefusion F Vaccine in Older Adults. N. Engl. J. Med..

[82] Papi A., Ison M.G., Langley J.M., Lee D.-G., Leroux-Roels I., Martinon-Torres F., Schwarz T.F., van Zyl-Smit R.N., Campora L., Dezutter N. (2023). Respiratory Syncytial Virus Prefusion F Protein Vaccine in Older Adults. N. Engl. J. Med..

[83] Walsh E.E., Falsey A.R., Scott D.A., Gurtman A., Zareba A.M., Jansen K.U., Gruber W.C., Dormitzer P.R., Swanson K.A., Radley D. (2022). A Randomized Phase 1/2 Study of a Respiratory Syncytial Virus Prefusion F Vaccine. J. Infect. Dis..

[84] Falsey A.R., Walsh E.E., Scott D.A., Gurtman A., Zareba A., Jansen K.U., Gruber W.C., Dormitzer P.R., Swanson K.A., Jiang Q. (2022). Phase 1/2 Randomized Study of the Immunogenicity, Safety, and Tolerability of a Respiratory Syncytial Virus Prefusion F Vaccine in Adults With Concomitant Inactivated Influenza Vaccine. J. Infect. Dis..

[85] Athan E., Baber J., Quan K., Scott R.J., Jaques A., Jiang Q., Li W., Cooper D., Cutler M.W., Kalinina E.V. (2024). Safety and Immunogenicity of Bivalent RSVpreF Vaccine Coadministered With Seasonal Inactivated Influenza Vaccine in Older Adults. Clin. Infect. Dis..

[86] Schwarz T.F., Hwang S.-J., Ylisastigui P., Liu C.-S., Takazawa K., Yono M., Ervin J.E., Andrews C.P., Fogarty C., Eckermann T. (2024). Immunogenicity and Safety Following 1 Dose of AS01E-Adjuvanted Respiratory Syncytial Virus Prefusion F Protein Vaccine in Older Adults: A Phase 3 Trial. J. Infect. Dis..

[87] Ferguson M., Schwarz T.F., Núñez S.A., Rodríguez-García J., Mital M., Zala C., Schmitt B., Toursarkissian N., Mazarro D.O., Großkopf J. (2024). Noninferior Immunogenicity and Consistent Safety of Respiratory Syncytial Virus Prefusion F Protein Vaccine in Adults 50–59 Years Compared to ≥60 Years of Age. Clin. Infect. Dis..

[88] Chen G.L., Mithani R., Kapoor A., Lu S., Asmar L.E., Panozzo C.A., Shaw C.A., Stoszek S.K., August A. (2022). 234. Safety and Immunogenicity of mRNA-1345, an mRNA-Based RSV Vaccine in Younger and Older Adult Cohorts: Results from a Phase 1, Randomized Clinical Trial. Open Forum Infect. Dis..

[89] Samy N., Reichhardt D., Schmidt D., Chen L.M., Silbernagl G., Vidojkovic S., Meyer T.P., Jordan E., Adams T., Weidenthaler H. (2020). Safety and Immunogenicity of Novel Modified Vaccinia Ankara-Vectored RSV Vaccine: A Randomized Phase I Clinical Trial. Vaccine.

[90] Aliprantis A.O., Shaw C.A., Griffin P., Farinola N., Railkar R.A., Cao X., Liu W., Sachs J.R., Swenson C.J., Lee H. (2021). A Phase 1, Randomized, Placebo-Controlled Study to Evaluate the Safety and Immunogenicity of an mRNA-Based RSV Prefusion F Protein Vaccine in Healthy Younger and Older Adults. Hum. Vaccines Immunother..

[91] Falsey A.R., Williams K., Gymnopoulou E., Bart S., Ervin J., Bastian A.R., Menten J., De Paepe E., Vandenberghe S., Chan E.K.H. (2023). Efficacy and Safety of an Ad26.RSV.preF–RSV preF Protein Vaccine in Older Adults. N. Engl. J. Med..

[92] Wagner A., Garner-Spitzer E., Jasinska J., Kollaritsch H., Stiasny K., Kundi M., Wiedermann U. (2018). Age-Related Differences in Humoral and Cellular Immune Responses after Primary Immunisation: Indications for Stratified Vaccination Schedules. Sci. Rep..

[93] Edelman R., Deming M.E., Toapanta F.R., Heuser M.D., Chrisley L., Barnes R.S., Wasserman S.S., Blackwelder W.C., Handwerger B.S., Pasetti M. (2020). The SENIEUR Protocol and the Efficacy of Hepatitis B Vaccination in Healthy older Persons by Age, Gender, and Vaccine Route. Immun. Ageing.

[94] Tahir A., Shinkafi S.H., Alshrari A.S., Yunusa A., Umar M.T., Hudu S.A., Jimoh A.O. (2024). A Comprehensive Review of Hepatitis B Vaccine Nonresponse and Associated Risk Factors. Vaccines.

[95] Versteegen P., Barkoff A.-M., Valente Pinto M., van de Kasteele J., Knuutila A., Bibi S., de Rond L., Teräsjärvi J., Sanders K., de Zeeuw-Brouwer M. (2022). Memory B Cell Activation Induced by Pertussis Booster Vaccination in Four Age Groups of Three Countries. Front. Immunol..

[96] Rack C., Almanzar G., Schäfer A., Völkl S., Dobler G., Mutterer A., Schmalzing M., Hick S., Steimer M., Jahn L. (2024). Immunogenicity of Tick-Borne-Encephalitis-Virus-(TBEV)-Vaccination and Impact of Age on Humoral and Cellular TBEV-Specific Immune Responses in Patients with Rheumatoid Arthritis. Vaccine.

[97] Stephenson I., Bugarini R., Nicholson K.G., Podda A., Wood J.M., Zambon M.C., Katz J.M. (2005). Cross-Reactivity to Highly Pathogenic Avian Influenza H5N1 Viruses after Vaccination with Nonadjuvanted and MF59-Adjuvanted Influenza A/Duck/Singapore/97 (H5N3) Vaccine: A Potential Priming Strategy. J. Infect. Dis..

[98] O’Hagan D.T., Ott G.S., De Gregorio E., Seubert A. (2012). The Mechanism of Action of MF59—An Innately Attractive Adjuvant Formulation. Vaccine.

[99] Nicolay U., Heijnen E., Nacci P., Patriarca P.A., Leav B. (2019). Immunogenicity of aIIV3, MF59-Adjuvanted Seasonal Trivalent Influenza Vaccine, in Older Adults ≥65 Years of Age: Meta-Analysis of Cumulative Clinical Experience. Int. J. Infect. Dis..

[100] Li A.P.Y., Cohen C.A., Leung N.H.L., Fang V.J., Gangappa S., Sambhara S., Levine M.Z., Iuliano A.D., Perera R.A.P.M., Ip D.K.M. (2021). Immunogenicity of Standard, High-Dose, MF59-Adjuvanted, and Recombinant-HA Seasonal Influenza Vaccination in Older Adults. Npj Vaccines.

[101] Squarcione S., Sgricia S., Biasio L.R., Perinetti E. (2003). Comparison of the Reactogenicity and Immunogenicity of a Split and a Subunit-Adjuvanted Influenza Vaccine in Elderly Subjects. Vaccine.

[102] Nicholson K.G., Abrams K.R., Batham S., Clark T.W., Hoschler K., Lim W.S., Medina M.-J., Nguyen-Van-Tam J.S., Read R.C., Warren F.C. (2011). Immunogenicity and Safety of a Two-Dose Schedule of Whole-Virion and AS03A-Adjuvanted 2009 Influenza A (H1N1) Vaccines: A Randomised, Multicentre, Age-Stratified, Head-to-Head Trial. Lancet Infect. Dis..

[103] Shi S., Zhu H., Xia X., Liang Z., Ma X., Sun B. (2019). Vaccine Adjuvants: Understanding the Structure and Mechanism of Adjuvanticity. Vaccine.

[104] Coffman R.L., Sher A., Seder R.A., Ivanova D.L., Thompson S.B., Klarquist J., Harbell M.G., Kilgore A.M., Lasda E.L., Hesselberth J.R. (2011). Adjuvant System AS03 Containing α-Tocopherol Modulates Innate Immune Response and Leads to Improved Adaptive Immunity. Vaccine.

[105] Sajkov D., Woodman R., Honda-Okubo Y., Barbara J., Chew D., Toson B., Petrovsky N. (2024). A Multiseason Randomized Controlled Trial of Advax-Adjuvanted Seasonal Influenza Vaccine in Participants With Chronic Disease or Older Age. J. Infect. Dis..

[106] Chen X., Zhao D., Liu X., He J., Lv R., Zhang J., Zhao Z., Wang L. (2023). Study on immune effect of Ganoderma lucidum spore polysaccharide as adjuvant of influenza vaccine. Jilin J. Chin. Med..

[107] Wang J., Ding Y., Chong K., Cui M., Cao Z., Tang C., Tian Z., Hu Y., Zhao Y., Jiang S. (2011). Recent Advances in Lipid Nanoparticles and Their Safety Concerns for mRNA Delivery. Front. Immunol..

[108] Siram K., Lathrop S.K., Abdelwahab W.M., Tee R., Davison C.J., Partlow H.A., Evans J.T., Burkhart D.J. (2022). Co-Delivery of Novel Synthetic TLR4 and TLR7/8 Ligands Adsorbed to Aluminum Salts Promotes Th1-Mediated Immunity against Poorly Immunogenic SARS-CoV-2 RBD. Vaccines.

[109] Zhang W.-Y., Zheng X.-L., Coghi P.S., Chen J.-H., Dong B.-J., Fan X.-X., Hallam J., Jones T., Alley J., Kohut M.L. (2024). Revolutionizing Adjuvant Development: Harnessing AI for next-Generation Cancer Vaccines. Front. Immunol..

[110] Coffman R.L., Sher A., Seder R.A. (2023). Vaccine Adjuvants: Putting Innate Immunity to Work. Immunity.

[111] O’Hagan D.T., Fox C.B. (2015). New Generation Adjuvants—From Empiricism to Rational Design. Vaccine.

[112] Reed S.G., Orr M.T., Fox C.B. (2013). Key Roles of Adjuvants in Modern Vaccines. Nat. Med..

[113] Coffman R.L., Sher A., Seder R.A., Ivanova D.L., Thompson S.B., Klarquist J., Harbell M.G., Kilgore A.M., Lasda E.L., Hesselberth J.R. (2023). Vaccine Adjuvant-Elicited CD8+ T Cell Immunity Is Co-Dependent on T-Bet and FOXO1. Cell Rep..

[114] Sanchez L., Nakama T., Nagai H., Matsuoka O., Inoue S., Inoue T., Shrestha A., Pandey A., Chang L.-J., De Bruijn I. (2023). Superior Immunogenicity of High-Dose Quadrivalent Inactivated Influenza Vaccine versus Standard-Dose Vaccine in Japanese Adults ≥ 60 Years of Age: Results from a Phase III, Randomized Clinical Trial. Vaccine.

[115] Ortiz de Lejarazu R., Martinón Torres F., Gil de Miguel Á., Díez Domingo J., Redondo Marguello E. (2021). High-dose trivalent influenza vaccine: Safety and immunogenicity. Rev. Esp. Quimioter..

[116] Jackson L.A., El Sahly H.M., George S., Winokur P., Edwards K., Brady R.C., Rouphael N., Keitel W.A., Mulligan M.J., Burton R.L. (2018). Randomized Clinical Trial of a Single versus a Double Dose of 13-Valent Pneumococcal Conjugate Vaccine in Adults 55 through 74 years of Age Previously Vaccinated with 23-Valent Pneumococcal Polysaccharide Vaccine. Vaccine.

[117] Anderson E.J., Rouphael N.G., Widge A.T., Jackson L.A., Roberts P.C., Makhene M., Chappell J.D., Denison M.R., Stevens L.J., Pruijssers A.J. (2020). Safety and Immunogenicity of SARS-CoV-2 mRNA-1273 Vaccine in Older Adults. N. Engl. J. Med..

[118] Kitamura S., Matsushita M., Komatsu N., Yagi Y., Takeuchi S., Seo H. (2020). Impact of Repeated Yearly Vaccination on Immune Responses to Influenza Vaccine in an Elderly Population. Am. J. Infect. Control.

[119] Zuo F., Abolhassani H., Du L., Piralla A., Bertoglio F., de Campos-Mata L., Wan H., Schubert M., Cassaniti I., Wang Y. (2022). Heterologous Immunization with Inactivated Vaccine Followed by mRNA-Booster Elicits Strong Immunity against SARS-CoV-2 Omicron Variant. Nat. Commun..

[120] Leroux-Roels I., Van Ranst M., Vandermeulen C., Abeele C.V., De Schrevel N., Salaun B., Verheust C., David M.-P., Kotb S., Hulstrøm V. (2024). Safety and Immunogenicity of a Revaccination With a Respiratory Syncytial Virus Prefusion F Vaccine in Older Adults: A Phase 2b Study. J. Infect. Dis..

[121] Kawakami K., Kishino H., Kanazu S., Toshimizu N., Takahashi K., Sterling T., Wang M., Musey L. (2016). Revaccination with 23-Valent Pneumococcal Polysaccharide Vaccine in the Japanese Elderly Is Well Tolerated and Elicits Immune Responses. Vaccine.

[122] Vukmanovic-Stejic M., Chambers E.S., Suárez-Fariñas M., Sandhu D., Fuentes-Duculan J., Patel N., Agius E., Lacy K.E., Turner C.T., Larbi A. (2018). Enhancement of Cutaneous Immunity during Aging by Blocking P38 Mitogen-Activated Protein (MAP) Kinase–Induced Inflammation. J. Allergy Clin. Immunol..

[123] Mannick J.B., Del Giudice G., Lattanzi M., Valiante N.M., Praestgaard J., Huang B., Lonetto M.A., Maecker H.T., Kovarik J., Carson S. (2014). mTOR Inhibition Improves Immune Function in the Elderly. Sci. Transl. Med..

[124] Hallam J., Jones T., Alley J., Kohut M.L. (2022). Exercise after Influenza or COVID-19 Vaccination Increases Serum Antibody without an Increase in Side Effects. Brain. Behav. Immun..

[125] Şevgin Ö., Özer S. (2024). Effect of Physical Exercise on Inactivated COVID-19 Vaccine Antibody Response in the Elderly. Hum. Antibodies.

[126] Papp G., Szabó K., Jámbor I., Mile M., Berki A.R., Arany A.C., Makra G., Szodoray P., Csiki Z., Balogh L. (2021). Regular Exercise May Restore Certain Age-Related Alterations of Adaptive Immunity and Rebalance Immune Regulation. Front. Immunol..

[127] Castro-Herrera V.M., Fisk H.L., Wootton M., Lown M., Owen-Jones E., Lau M., Lowe R., Hood K., Gillespie D., Hobbs F.D.R. (2021). Combination of the Probiotics Lacticaseibacillus Rhamnosus GG and Bifidobacterium Animalis Subsp. Lactis, BB-12 Has Limited Effect on Biomarkers of Immunity and Inflammation in Older People Resident in Care Homes: Results From the Probiotics to Reduce Infections iN CarE Home reSidentS Randomized, Controlled Trial. Front. Immunol..

[128] Fernández-Ferreiro A., Formigo-Couceiro F.J., Veiga-Gutierrez R., Maldonado-Lobón J.A., Hermida-Cao A.M., Rodriguez C., Bañuelos O., Olivares M., Blanco-Rojo R. (2022). Effects of Loigolactobacillus Coryniformis K8 CECT 5711 on the Immune Response of Elderly Subjects to COVID-19 Vaccination: A Randomized Controlled Trial. Nutrients.

